# Variation of cataract surgery costs in four different graded providers of China

**DOI:** 10.1186/1471-2458-10-543

**Published:** 2010-09-09

**Authors:** Jiahua Fang, Xinghua Wang, Zhende Lin, Jihua Yan, Ye Yang, Jingbo Li

**Affiliations:** 1 Department of Ophthalmology, First Hospital of Jingzhou, Yangtze University, Jingzhou 434000, China; 2 Department of Ophthalmology, Union Hospital, Tongji Medical College, Huazhong University of Science and Technology, Wuhan 430022, China; 3 State Key Laboratory of Ophthalmology, Zhongshan Ophthalmic Centre, Sun Yat-sen University, Guangzhou 510060, China; 4 Jingshan County Hospital, Jingshan 431800, Hubei Province, China

## Abstract

**Background:**

China has the largest population of cataract patients in the world. However, the cataract surgery rate per million remains low in China. We carried out a survey on costs of cataract surgery from four different graded providers in China and analyzed differences in cost among these clinics.

**Methods:**

1,189 patients were recruited for the study in four eye clinics, located in two provinces, Guangdong province in southern China and Hubei province in central China. The average cost of each cataract surgery episode was calculated including cost of intraocular lens, cost of drugs and facility cost. We also collected information on reimbursement and disposable annual income of local residents.

**Results:**

Mean total cost per cataract intervention of four different providers varied considerably, ranging from US$ 1,293 in Union Hospital to US$ 536 in Jingshan County Hospital. In all providers, except for Jingshan County Hospital, the cost exceeded annual disposable income of local rural residents. As to the proportion of patients with reimbursement, the figure for Union Hospital was only 36%, while for other three clinics it was more than 60%. There was a significant difference between mean reimbursement ratios, with the highest ratio in Zhongshan Ophthalmic Center being 71%.

**Conclusions:**

Significant differences in costs of cataract surgery were found among the 4 different graded providers. A part of the cost was borne by patients. Proportion of patients with reimbursement and mean reimbursement ratios were higher in economically developed regions than in economically developing regions. Much more financial support should be directed into the rural New Cooperative Medical Scheme to raise the reimbursement ratio in rural China.

## Background

Cataract is a leading cause of blindness around the world. Approximately 18 million people worldwide are blind from bilateral mature cataracts[1]. In developing countries 50-90% of all blindness cases are caused by cataracts. As the world's largest developing country, China is a country with nearly 1.4 billion population, among which there are 2.5 million cataracts with an annual increase of 400 thousand patients[2]. Removing the cataract surgically and replacing the natural lens with a synthetic intraocular lens (IOL) remain the only effective treatment for cataracts. The cataract surgery rate (CSR, the number of cataract operations performed per million populations per year) is about 5700 in the United States[3], but only about 4500 in India[4], and as low as 200 in some developing countries[5,6]. However, the CSR may vary between provinces in China, such as 1197 in Beijing, 1548 in Shanghai, 953 in Tibet, 608 in Qinghai; but only 196 in Guizhou, 216 in Chongqing[7]. In the developing world, China and India are the two most populous countries on earth and are at a roughly comparable stage of economic development, while the CSR is considerably lower in China than in India. Most of the patients who suffer from reversible blindness due to mature cataracts go untreated in China; as a result, the number of blind patients is growing every year.

From a public health perspective, it is clear that senile cataract remains a leading cause of blindness in China and our population is experiencing a "backlog" of cataract. Poor services that are inaccessible, inappropriate, or unaffordable will not be utilised and people with unoperated cataract will accumulate to form a surgical backlog[3]. To reduce the backlog, the number of cataract operations performed each year must at least equal the incidence of operable cataract. A cataract screening program, including a total of 27 screening groups, was conducted in the rural Chaoshan area of eastern Guangdong province between March 2008 and March 2009. Among 24 screening group subjects refusing cataract surgery, the most common reasons were concern about costs (60.4%), and unwillingness to undergo surgery due to age (22.9%)[8]. Therefore, the cost of cataract surgery may be an important factor that influences the amount of cataract surgery[8-10].

Cataract is a common public health problem in patients above 50 years old in China. Although the magnitude of the problem is so large, little is known about the costs of cataract surgery or variation in costs among different providers in China. Moreover, the patterns of cataract surgery, economic status of population and reimbursement ratios have changed greatly in the past few years, but little is known about the relation between costs of surgery and disposable annual income of local residents. Average costs incurred by cataract surgery alone in the United Kingdom were about US$620 in 2004 dollars, but another research demonstrated that the cost is US$3461, approximately 5.6 times of the former one. This is because of the difference between direct and indirect costs [11]. In 2005, the mean total costs per cataract intervention from nine European countries(Denmark, England, France, Germany, Hungary, Italy, the Netherlands, Poland, and Spain) were €714, ranging from €318 to €1087(€1 = US$1.18 for 2005)[12]. Cataract surgery is considerably less expensive in Europe than in the United States with the mean cost of surgery totaled US$2525[13,14].

In order to achieve a higher CSR, it is necessary to investigate the barriers that may be preventing patients from seeking surgery in China. The aim of this survey performed in four different graded providers of China was to collect information on costs of cataract surgery and to compare the overall cost of surgery with disposable annual income of local residents. It is also hoped that the survey will provide a valuable source of data for developing new strategies for the prevention and treatment of blindness caused by cataract.

## Methods

**Figure 1:**
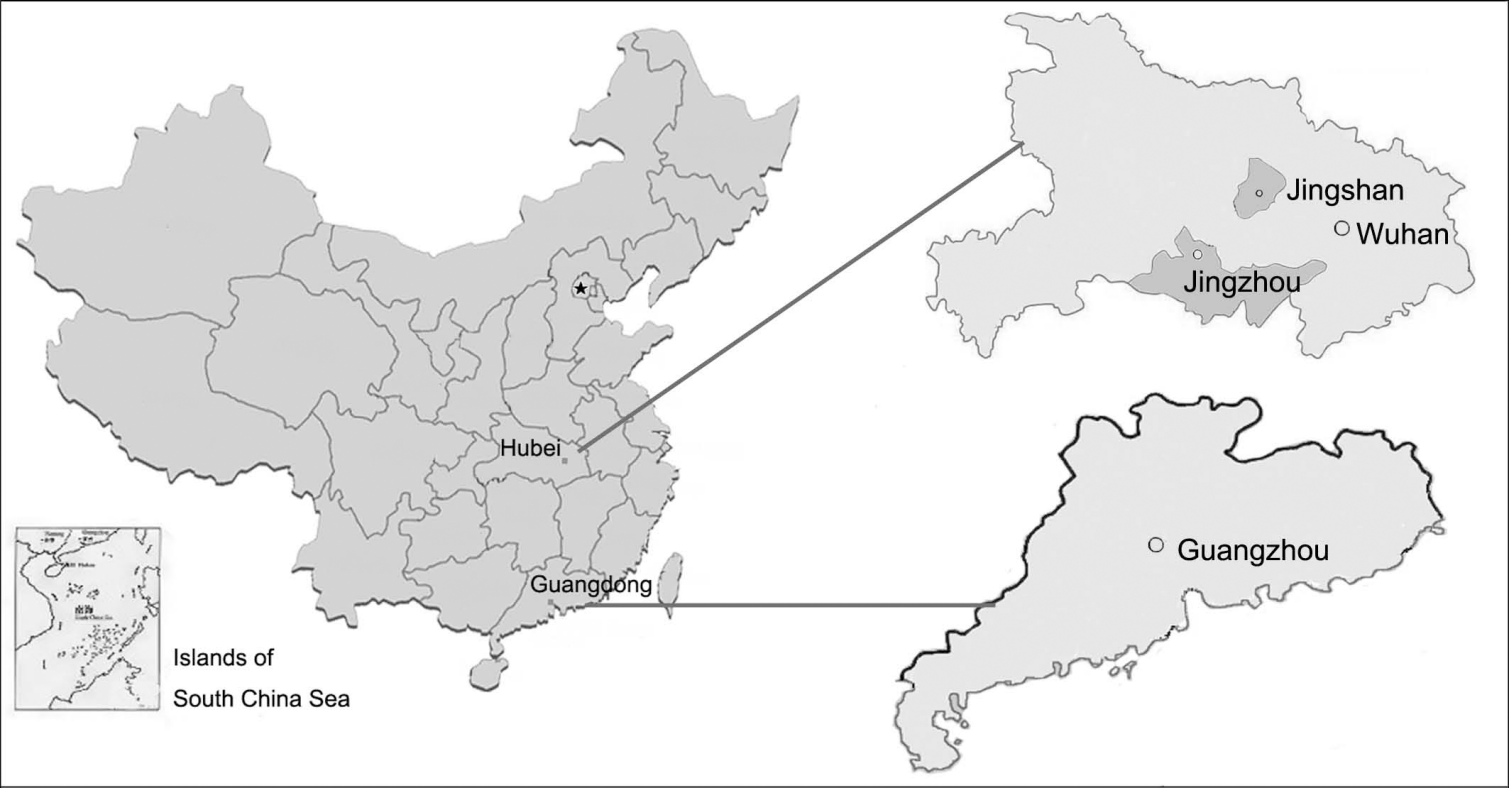
**Locations of four different graded providers in two provinces of China**.

Subjects with age-related cataract for this study were recruited from four different graded providers in two provinces (Figure 1). Zhongshan Ophthalmic Center (ZOC) is the largest eye hospital in China, located in Guangdong province, one of the most economic developed regions in China. The other three eye clinics from three general hospitals are located in Hubei province in central China. Union Hospital (UH) is located in Wuhan, capital of Hubei province. First Hospital of Jingzhou (FHJ) is located in Jingzhou, one medium-sized city. Jingshan County Hospital (JCH) is located in Jianshan County, which is a typical agricultural county with a total population of 640,000, 69% are farmers. These four eye clinics represent four different size and grade respectively in China. These clinics are described in details in Table 1.

**Table 1:** Characteristics of the four clinics and cataract surgery

Clinic	ZOC	UH	FHJ	JCH
Inhabitants in catchment area for cataract surgery	96,380,000	57,200,000	6,450,000	640,000
Number of cataract surgeries in 2009	13,348	866	510	353
Number of cataract surgeries during study month (November 2009)	1,102	82	46	28
Number of patients	1,038	78	45	28
Median age of patients(y)	71	66	69	67
Male (%)	47%	43%	52%	54%
Number of patients from rural region	126(12%)	5(6%)	5(11%)	12(43%)
Percentage of phaco-procedures during study month	99%	99%	98%	100%
Average length of stay (days)	1	8	9	1
Proportion of cases with foldable lens	100%	99%	0%	7%
Number of patients with reimbursement	766(74%)	28(36%)	30(67%)	20(71%)
Mean reimbursement ratio	71%	50%	45%	48%
Number of cataract surgeons in 2009	17	4	2	0

The average cost of a cataract extraction at each clinic during the month of November 2009 was studied. The cost of a cataract surgery episode including one pre-operative visit, the surgery itself and one post-operative visit was calculated in three sections as follows: cost of intraocular lens, cost of drugs and facility cost. Facility cost included laboratory test expenses, nursing expenses, check fees, costs of equipment depreciation, and the costs of one preoperative and one postoperative visit, etc. The check fees included the costs of ultrasonic examination, intraocular pressure measurement and slit lamp examination. Operating microscopes and phacoemulsifier were the main equipments used in cataract surgery. In the case of JCH, the cost of physician and his or her equipment charges went under facility costs. Costs such as travel costs and costs of complications of surgery were excluded. Costs were based on hospital finance department data. In addition to the costs for one episode of cataract surgery, each provider was asked to provide information on the reimbursement fees they received for performing the service.

Disposable annual incomes of local urban and rural residents were acquired from the websites of National and Provincial Bureau of Statistics (Data from Guangdong province available from http://www.gdstats.gov.cn/tjgb/t20100225_74438.htm; Hubei province available from http://www.stats-hb.gov.cn/structure/xxgk/tjgb/qstjgbzw_185369_1.htm; Jingzhou city available from http://www.stats-hb.gov.cn/structure/xxgk/tjgb/sztjgbzw_187478_1.htm; Jingshan County available from http://www.jingshan.gov.cn/article/2010/0120/article_8100.html; Accessed on 5 July 2010). All costs were collected in Renminbi (RMB) and converted to US dollars (US$) from an exchange rate of US$ 1 = RMB 6.8. In the study, we used the principles outlined in the Helsinki declaration. The study was approved by the ethical committees of Yangtze University, Huazhong University of Science and Technology, Sun Yat-sen University and Jingshan County.

## Results

A total of 1,189 patients were recruited by 4 eye clinics: 1,038 patients from ZOC, 78 patients from UH, 45 patients from FHJ and 28 patients from JCH. Some patients underwent bilateral surgeries, thus a total of 1,264 operations were performed in four clinics (Table 1). The number of cataract surgeries during November 2009 performed in ZOC was ten times more than in the other three hospitals. The proportion of patients from rural areas in JCH reached 43%, while for the other three bigger hospitals was very low. Phacoemulsification procedure was the primary surgery used in all four hospitals and extracapsular cataract extraction (ECCE) was seldom used in these hospitals. The proportion of ECCE in these clinics was less than 2 percent. The average length of stay in ZOC and JCH was one day, but more than one week in UH and FHJ. Characteristics of the four clinics and cataract surgeries were shown in Table 1.

**Figure 2:**
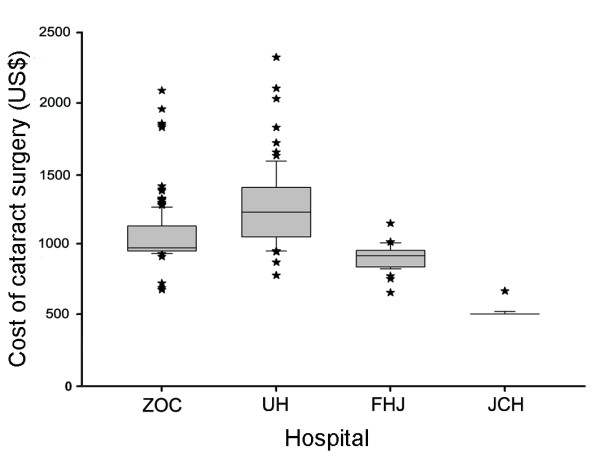
**Boxplots showing the average cost for each cataract surgery episode from four different graded providers during November 2009**. Horizontal lines in each box plot represent (bottom to top) 10th, 25th, 50th (median), 75th, and 90th percentiles. Values above the 90th and below the 10th percentile are plotted as stars. The abbreviations used are: ZOC, Zhongshan Ophthalmic Center; UH, Union Hospital; FHJ, First Hospital of Jingzhou; JCH, Jingshan County Hospital.

The average total costs for one cataract surgery episode in four clinics were 1,089 US$ (median, 973 US$; range, 713 US$ to 2,122 US$) in ZOC, 1,293 US$ (median, 1,236 US$; range, 794 US$ to 2,345 US$) in UH, 925 US$ (median, 930 US$; range, 681 US$ to 1,170 US$) in FHJ and 536 US$ (median, 526 US$; range, 526 US$ to 685 US$) in JCH. The costs of cataract surgery in two provincial hospitals were twice as many as in JCH (Figure 2). There was no qualified cataract surgeon in JCH, just like the majority of county hospitals in China. Qualified surgeons from provincial capital carried portable phacoemulsifier to JCH to perform cataract surgery once or twice a month and received a medical consultation fee.

**Figure 3:**
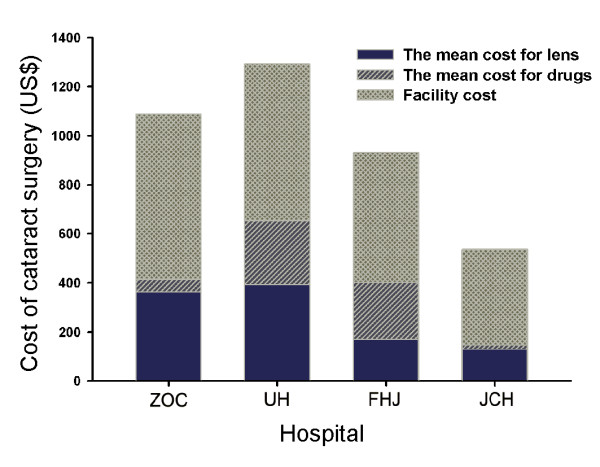
**Components of cataract surgery costs in four different graded providers**. The abbreviations used are: ZOC, Zhongshan Ophthalmic Center; UH, Union Hospital; FHJ, First Hospital of Jingzhou; JCH, Jingshan County Hospital.

**Figure 4:**
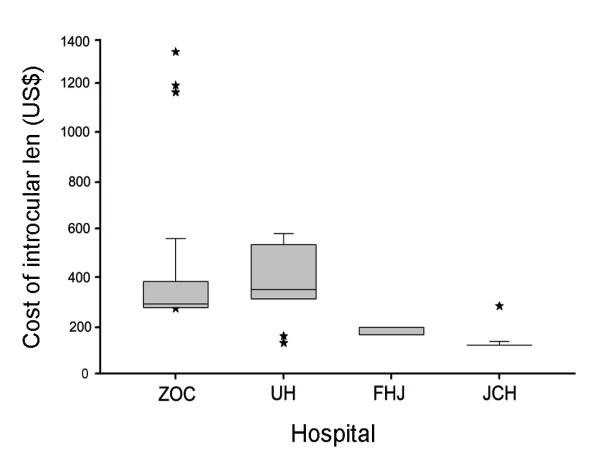
**The average cost for intraocular lens from four different graded providers in November 2009**. Horizontal lines in each box plot represent (bottom to top) 10th, 25th, 50th (median), 75th, and 90th percentiles. Values above the 90th and below the 10th percentile are plotted as stars. The abbreviations used are: ZOC, Zhongshan Ophthalmic Center; UH, Union Hospital; FHJ, First Hospital of Jingzhou; JCH, Jingshan County Hospital.

The different components of cataract surgery costs identified so far are summarized in Figure 3. The mean costs of intraocular lens in four clinics were 364 US$(median, 289 US$; range, 273 US$ to 1,379 US$) in ZOC, 392 US$(median, 347 US$; range, 127 US$ to 579 US$) in UH, 170 US$(median, 162 US$; range, 162 US$ to 191 US$) in FHJ and 129 US$(median, 118 US$; range, 118 US$ to 279 US$) in JCH(Figure 4). Large differences were found among the 4 clinics. Multi-focal intraocular lens or adjustable intraocular lens (24/1108, 2.16%) cost more than 1,100 US$, which was only used in ZOC. All patients in ZOC were implanted foldable lens, but rigid lens were used in FHJ and JCH. The cost of the cheapest lens used was 118 US$ in JCH. There were 16 different types of lens used in ZOC, but only 2 types of lens used in FHJ and JCH.

The major part of cost was the use of antibiotics by intravenous infusion in UH and FHJ. Cataract surgeries in the two hospitals were performed via corneal limbal incision, and patients had been given antibiotics once a day after operation for 3 to 7 days aiming to prevent infection. Then, the length of stay was one week or so. The average cost of antibiotics for each patient was more than 200 US$. However, a clear corneal incision was used in ZOC and JCH, and patients stayed for one day without intravenous infusion of drug. The main cost for drugs in the two hospitals was the fee for eyedrops.

The number of patients with reimbursement was 766(74%) in ZOC, 28(36%) in UH, 30(67%) in FHJ and 20(71%) in JCH. Not all patients received reimbursement. The patients had to pay first, and then were "reimbursed" by the insurance company or rural New Cooperative Medical Scheme (NCMS). In ZOC, the mean reimbursement ratio (reimbursement/cost) was approximately 71%, ranging from 30% to 95%. The cost of the surgery didn't vary according to whether the patient had insurance. As a part of urban residents didn't get health insurance and reimbursement ratio for rural residents was quite low, a part of the cost was paid by the patients directly.

**Figure 5:**
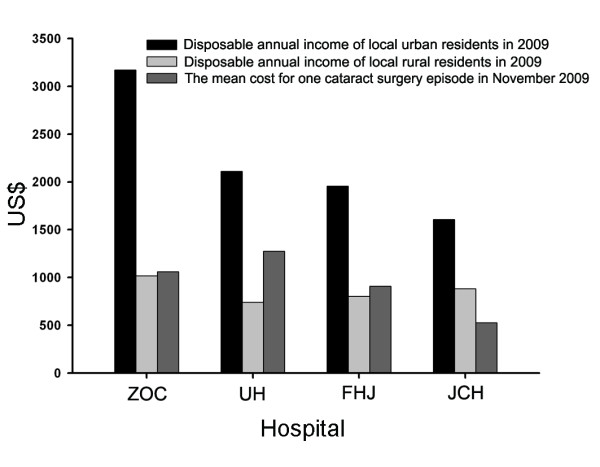
**Comparison among disposable annual income of local urban residents in 2009, disposable annual income of local rural residents in 2009 and the average cost for each cataract surgery episode in November 2009**. The abbreviations used are: ZOC, Zhongshan Ophthalmic Center; UH, Union Hospital; FHJ, First Hospital of Jingzhou; JCH, Jingshan County Hospital.

We compared the mean cost of one cataract surgery episode with disposable annual income of local residents in 2009(Figure 5). In Guangdong province, the Gross Domestic Product (GDP) per capita in 2009 was 5,992US$, with disposable annual income of local urban residents 3,173 US$ and rural residents 1,016 US$, respectively. However in Hubei province, the disposable annual income of local urban residents was 2,113 US$ and rural residents 740 US$, respectively. In all providers except for JCH, the cost exceeded annual disposable income of local rural residents. Proportion of patients with reimbursement and mean reimbursement ratios in ZOC were higher than that in three hospitals in Hubei province, which was related with local economic level.

## Discussion

This study investigated the costs of cataract surgery from four different graded providers in two provinces of China and analyzed the variation in costs. The results of this study may not be necessarily representative of patients presenting in the other regions of China. The mean total cost for one cataract surgery episode from two hospitals in capital cities, ZOC and UH, was more than 1,000 US$. The lowest cost was from JCH, approximately 536 US$. In all providers except for JCH, the cost was more than annual disposable income of local rural residents. Especially in the UH, the cost was twice as many as annual disposable income of local rural residents.

Except the proportion of patients with reimbursement in UH was at only 36%, the proportion in the other three hospitals was more than 60%. There were significant variations in reimbursement ratio from different regions. The mean reimbursement ratio was higher in ZOC than in three hospitals in Hubei province. The more economy develops, the higher reimbursement ratio is. Urban patients can be reimbursed at about 40-95% from social medical insurance. Seven years ago, rural patients must pay the cost all by themselves[15], but at present, more than 90% rural residents can be reimbursed due to the attendance of NCMS. However, the ratio of reimbursement for rural patients was approximately 30%, which was significantly lower than urban patients[16].

China's economic development is characterized by wide geographic disparities between the coastal regions and the central/western regions. Two thirds of China's population, or around 900 million people, live in rural regions that are largely under-developed and have received few medical services. China had developed a successful health insurance system (Cooperative Medical Scheme, CMS) in the rural areas since the 1950s. Unfortunately, CMS collapsed during the shift towards a market economy at the end of the 1970s[17]. The New Cooperative Medical Scheme (NCMS) is a 'voluntary' and heavily subsidized program established in 2003 to reduce the risk of catastrophic health spending for rural residents in China [15,18,19]. The scheme coverage was 95.9% in Shandong province and 88.0% in Ningxia province in 2006[19], and 85.9% of the total rural population by the end of 2007[16]. However, the reimbursement ratio of the scheme was only around 30% of inpatient expenditure.

The relatively poor economic condition in rural areas is a major issue influencing the feasibility of performing cataract surgeries [20]. In 2001, Helen Keller International (HKI) initiated free cataract testing and a low-price, high-quality cataract surgery program in rural areas in south China [9]. Surgery was subsidized by HKI and priced at 66US$. A total of 80% of those surveyed stated that they were willing to pay something for surgery, but only 56% of these respondents stated a willingness to pay amount of 66US$ or more. Blind subjects were significantly more likely to pay anything for surgery, but would pay on average 32US$. The patients suffering from cataract were mostly older patients, and many of them were no longer in control of their family's income. Compared with the rural annual income per capita, the expensive cost for cataract surgery may be an important contributing factor for the low CSR in rural China.

In our survey, the number of patients from rural regions was significantly lower than that from urban regions. Jingshan County located in central China owns 640,000 people, with approximately 440,000 people living in rural areas. Our data showed that the cataract surgery rate (CSR) per million in Jingshan County was 552, about one-tenth of that in the United States[21]. Therefore, there is apparent potential for an increase of CSR in China, especially in rural areas.

A large variation in costs of intraocular lens was found in four providers. A foldable intraocular lens (IOL) is more expensive than an equivalent rigid IOL. In ZOC, all patients were implanted with foldable intraocular lens. The variation of total costs among patients mainly came from the cost of different IOL types. Costs of multi-focal intraocular lens or adjustable intraocular lens were more than 1,100 US$, which were too expensive to be reimbursed; thereby most patients chose single focus intraocular lens. In three hospitals of Hubei province, neither multifocal nor adjustable intraocular lens was used. All four providers used imported lens. Although there are cheaper domestic lens, none of domestic lens was used in these hospitals because patients thought that the quality of imported lens was better than domestic lenses.

Under the current conditions, phacoemulsification and small incision surgery have allowed cataract surgery to be conducted on one-day-case basis, and then it is feasible to use eyedrops to prevent infection. A large part of cost was spent on the usage of antibiotics by vein injection in UH and FHJ. Therefore, using antibiotic eyedrops instead of intravenous antibiotics would decrease 20% cost of cataract intervention.

There are no qualified surgeons of cataract surgery in Jingshan County Hospital. In China, there are around 2,200 counties like Jingshan County; and the overwhelming majority of county hospitals have neither qualified surgeons nor expensive equipments for phacoemulsification. Qualified surgeons from provincial capital carried portable phacoemulsification equipments to county hospitals and then performed cataract surgeries once or twice a month, which was a common health care activity. It is obvious that the phacoemulsification procedure is more expensive than ECCE. Manual small incision cataract surgery (MSICS) is a modified form of ECCE performed through a 6.5 to 7.0 mm sclerocorneal tunnel [22]. As compared with phacoemulsification, MSICS is almost as effective and more economical [10,23,24]. The advantages of MSICS as a low-cost "equally effective" technique make it an alternative, especially in rural regions in China. There are severe shortages of skilled cataract surgeons in the majority of county hospitals in China; thereby it is essential to train qualified surgeons for these hospitals.

Most of Chinese elderly population is rural-dwelling and cataract is the leading cause of blindness and low vision in this group. Approximately 66% of the cataract operations were conducted in county hospitals with limited eye care services and 34% in specialized and provincial center [20]. Because of the low benefit level and low reimbursement ratio, patients had to face a very high financial burden even after NCMS reimbursement[17]. Moreover, the shortage of cataract care specialists in county hospitals is also a barrier for many patients who need cataract surgery. Consequently, efforts should be made to increase the financing of health care and to train qualified surgeons for county hospitals.

Finally, the data from four different graded providers were provided in the results, which were of potential use to blindness prevention programs. However, there was no a statistical point of view in our report, and the selected four places could not fully represent the status of other areas in China. Further work is needed to explore the cost-effectiveness of cataract surgery in different graded provided in China.

## Conclusions

Significant differences in costs of cataract surgery were found among the 4 different graded providers. From the survey, several key messages should be drawn. Firstly, patients from rural areas have many difficulties in paying for surgery cost. Much more financial support should be directed into the rural NCMS to raise the reimbursement ratio. Secondly, more efforts are needed to train qualified surgeons for county hospitals. Lastly, the usage of cheaper IOL and fewer drugs in cataract surgery will lead to lower cost that contributes to increase the CSR in China.

## Competing interests

The authors declare that they have no competing interests.

## Authors' contributions

JHF participated in the design of the survey, undertook the collection of data and drafted the manuscript. XHW undertook the collection of data and assisted in drafting the manuscript. JHY, YY and JBL undertook the collection of data. ZDL participated in the design of the survey. All authors read and approved the final manuscript.

## Pre-publication history

The pre-publication history for this paper can be accessed here:

http://www.biomedcentral.com/1471-2458/10/543/prepub
